# Comparison of prostate verification with implanted gold markers in tissue surrounding the prostate and pelvic bony anatomy for external beam radiation therapy following low-dose-rate brachytherapy: a prospective clinical trial

**DOI:** 10.1093/jrr/rraa063

**Published:** 2020-08-18

**Authors:** Tomoya Kaneda, Toshio Ohashi, Takashi Hanada, Koji Takenaka, Shuichi Nishimura, Masanori Sakayori, Shinya Sutani, Tetsuo Momma, Naoyuki Shigematsu

**Affiliations:** Department of Radiology, Keio University School of Medicine, 35 Shinanomachi, Shinjuku-ku, Tokyo 160-8582, Japan; Department of Radiology, National Hospital Organization Saitama Hospital, 2-1 Suwa, Wako, Saitama 351-0102, Japan; Department of Radiology, Keio University School of Medicine, 35 Shinanomachi, Shinjuku-ku, Tokyo 160-8582, Japan; Department of Radiology, National Hospital Organization Saitama Hospital, 2-1 Suwa, Wako, Saitama 351-0102, Japan; Department of Radiology, Keio University School of Medicine, 35 Shinanomachi, Shinjuku-ku, Tokyo 160-8582, Japan; Department of Radiology, National Hospital Organization Saitama Hospital, 2-1 Suwa, Wako, Saitama 351-0102, Japan; Department of Radiology, Keio University School of Medicine, 35 Shinanomachi, Shinjuku-ku, Tokyo 160-8582, Japan; Department of Radiology, Keio University School of Medicine, 35 Shinanomachi, Shinjuku-ku, Tokyo 160-8582, Japan; Department of Radiology, National Hospital Organization Saitama Hospital, 2-1 Suwa, Wako, Saitama 351-0102, Japan; Department of Radiology, Keio University School of Medicine, 35 Shinanomachi, Shinjuku-ku, Tokyo 160-8582, Japan; Department of Urology, National Hospital Organization Saitama Hospital, 2-1 Suwa, Wako, Saitama 351-0102, Japan; Department of Radiology, Keio University School of Medicine, 35 Shinanomachi, Shinjuku-ku, Tokyo 160-8582, Japan

**Keywords:** prostate cancer, brachytherapy, external beam radiation therapy, fiducial marker

## Abstract

We aimed to investigate whether gold marker implantation in the tissue surrounding the prostate could accurately monitor setup errors during external beam radiation therapy (EBRT) following low-dose-rate (LDR) brachytherapy. Thirty-eight patients had confirmed intermediate- or high-risk prostate cancer and received EBRT following LDR brachytherapy. In >175 computed tomography imaging sessions, the average values of the weekly setup error during EBRT to the prostate centroid at the time of gold marker matching in the surrounding tissue of the prostate and pelvic bone matching were measured and then compared using the Wilcoxon signed-rank test. Gold marker matching in the surrounding tissue of the prostate estimated setup errors better than those estimated by bone matching (3D displacement = 2.7 ± 2.0 vs 3.8 ± 2.6 mm, *P* < 0.01). Overall, the standard deviation of systematic (Σ) and random (σ) setup error was lower with gold marker matching than with bone matching (3D displacement = 1.8 and 1.1 mm vs 2.1 and 1.6 mm, respectively). With gold marker matching, the setup error of the position of the prostate centroid was smaller, and the optimal setup margin was lower than that with bone matching (2Σ + 0.7σ and 2.5Σ + 0.7σ of 3D displacement = 4.3 and 5.2 mm vs 5.3 and 6.4 mm, respectively). This high-precision radiotherapy approach placing gold markers in the surrounding tissue of the prostate can allow more accurate setup during EBRT following LDR brachytherapy.

## INTRODUCTION

Delivery of external beam radiation therapy (EBRT) can be compromised by the daily setup as well as motion of the prostate during treatment [1–5]. Such errors can affect both the setup margin (interfractional and intrafractional setup errors) and the internal margin (organ motion errors). The close proximity of the rectum to the prostate dictates careful monitoring of prostate position during EBRT. Monitoring can minimize rectal toxicity and improve clinical outcomes [6, 7]. Minimizing setup errors is critically important in EBRT.

Conventional correction protocols for positioning of the patient during treatment of prostate cancer are offline when portal imaging based on matching the bony anatomy or skin markers is used [8–10]. Dependence on the use of external markers such as markings on the skin or reliance on the alignment of bony anatomy does not result in precise positioning of the prostate. The bony anatomy does not accurately represent the prostate position, and daily shifts in the position of the prostate caused by filling of organs such as the bladder and the rectum can be significant during EBRT fractions [10, 11]. Prostate displacement relative to the bony anatomy can be significant during EBRT fractions, or even within the same fraction. These factors can make accurate delivery of EBRT to prostate cancer difficult. Monitoring of localization uncertainties of the prostate, including changes in the position of the prostate or patient, is therefore of critical importance.

**Fig. 1.**
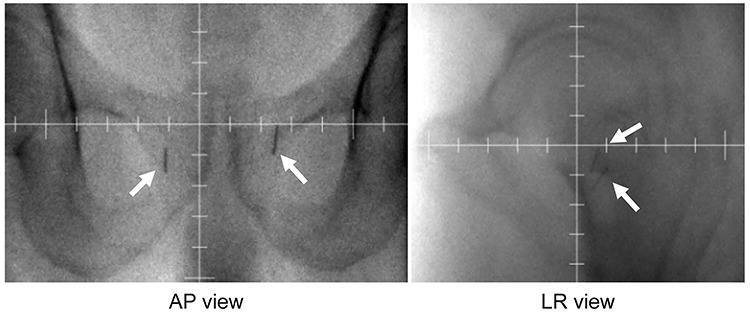
Linac 2D MV imaging (AP/LR view) showing the implantation of gold markers (thick arrows) in the surrounding tissue of the prostate. Implanted seeds in the prostate are invisible with MV imaging.

Implantation of gold markers as fiducials for prostate positioning during EBRT is reported to be a superior method in comparison to the use of bony anatomy for alignment. The use of implanted fiducial markers provides a more accurate estimate of prostate localization and in turn provides better estimates for planning target volume (PTV) margins [1, 12–16]. Regarding location verification of EBRT following low-dose-rate (LDR) brachytherapy by implanting permanent radioactive seeds, some seed models cannot be seen in the prostate with megavoltage (MV) imaging [17]. In our hospital, daily image-guided radiation therapy (IGRT) was only performed in linear accelerator (Linac) MV imaging because a kilovoltage (kV) imaging device was not attached (Fig. 1). Therefore, we cannot perform prostate verification by seed matching because MV imaging cannot detect seeds. If placing a gold marker in the prostate gland, the gold marker can cause the seeds to shift and disturb seed implant dose distributions [18]. To resolve these problems, we recently devised a method of inserting gold markers in the surrounding tissue of the prostate for IGRT without shifting the seeds and disturbing seed implant dose distributions. However, to our knowledge, there have been no clinical trials examining setup error with gold markers during EBRT following LDR brachytherapy. In fact, implantation of markers in the surrounding tissue of the prostate as an image-guiding technique during EBRT has not been fully explored.

The aims of the present study were to show the new IGRT method by gold marker matching in MV verification without using seed matching for EBRT following LDR brachytherapy and to investigate gold marker implantation in the surrounding tissue of the prostate using this technique to monitor setup errors and the optimal setup margin during EBRT.

## MATERIALS AND METHODS

### Patients

This prospective clinical trial (UMIN trial ID: UMIN000011078) was conducted at the National Hospital Organization Saitama Hospital between 1 July 2013 and 30 June 2014. Ethics approval for a human study was obtained from the institutional review board before this study was initiated. All participants signed a protocol-specific informed consent form in accordance with the institutional guidelines.

A total of 38 patients with localized prostate cancer receiving ^125^I brachytherapy combined with EBRT participated in this study. The following risk factors related to prostate cancer were assessed: serum levels of prostate-specific antigen (PSA), Gleason score and tumor-node-metastasis stage. The patients were divided into low-risk (T1-2a: PSA < 10 ng/ml and Gleason score ≤ 6), intermediate-risk (T2b: PSA 10–20 ng/ml or Gleason score = 7) and high-risk (T2c-3: PSA > 20 ng/ml or Gleason score ≥ 8) groups. Patients in the intermediate- to high-risk groups and those in the low-risk group with positive core needle biopsy rates >33% received the combined therapy. Before the initiation of EBRT, 24 patients (63%) were treated with neoadjuvant hormonal therapy (Table 1).

**Table 1:** Clinical characteristics of the patients

Factor	Value
Number of patients	38
Age (years), median (range)	70 (52–79)
Risk category, *n* (%)	
Low	1 (2.6)
Intermediate	24 (63.2)
High	13 (34.2)
EBRT technique	
3D-CRT	20
IMRT	18
Hormone therapy	24 (63.2)

3D-CRT = 3D conformal radiation therapy.

### Treatment technique

The loose or linked seeds (Brachysource; CR Bard, Covington, GA, USA) were implanted using an interactive ultrasound-guided technique with a peripheral loading pattern [19]. The PTV was defined as the entire prostate. The prescribed dose to the PTV was 110 Gy for intraoperative planning using the VariSeed 8.0 planning system (Varian Medical Systems, Inc., Palo Alto, CA, USA). All patients received a median EBRT dose of 45 Gy at 1.8 Gy per fraction, using 10-MV photons delivered by 3D conformal radiation therapy or intensity-modulated radiation therapy with a Linac (ONCOR Impression Plus; Siemens Medical Solutions, Erlangen, Germany) at 1 month after implantation. During EBRT, daily IGRT was conducted by 2D MV matching [anterior–posterior (AP)/left–right (LR)] using gold marker matching (Fig. 1). An immobilization device was not used and pretreatment (e.g. bladder filling and defecation) was performed.

### Marker implantation

**Fig. 2.**
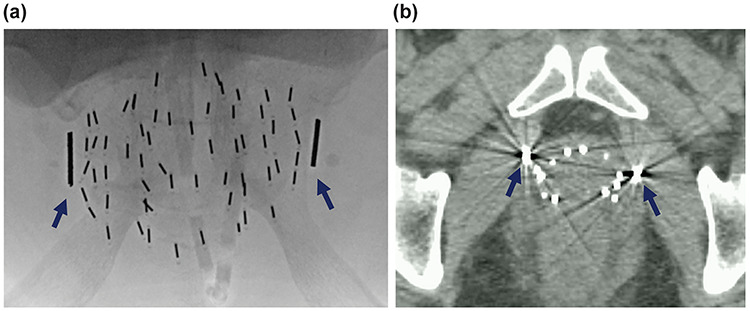
Pelvic radiography (**a**) and axial computed tomography scan (**b**) showing the implantation of gold markers (thick arrows) in the surrounding tissue of the prostate.

After seed treatment, radio-opaque fiducial markers consisting of a linear gold marker (10 mm length and 1.1 mm diameter, Visicoil; RadioMed, Bartlett, TN, USA) were embedded one at a time. The two markers were placed in both external sides adjacent to the prostate (Fig. 2). The gold marker was inserted using a combination of X-ray fluoroscopy and transrectal ultrasonography, which allowed observation of the position relative to the prostate. A course of preventive antibiotic treatment was administered and consisted of intravenous cefotiam (1 g twice daily on implant day and 1 day after implantation), followed by oral administration of levofloxacin (500 mg/day) for 7 days.

### Imaging and position matching

**Fig. 3.**
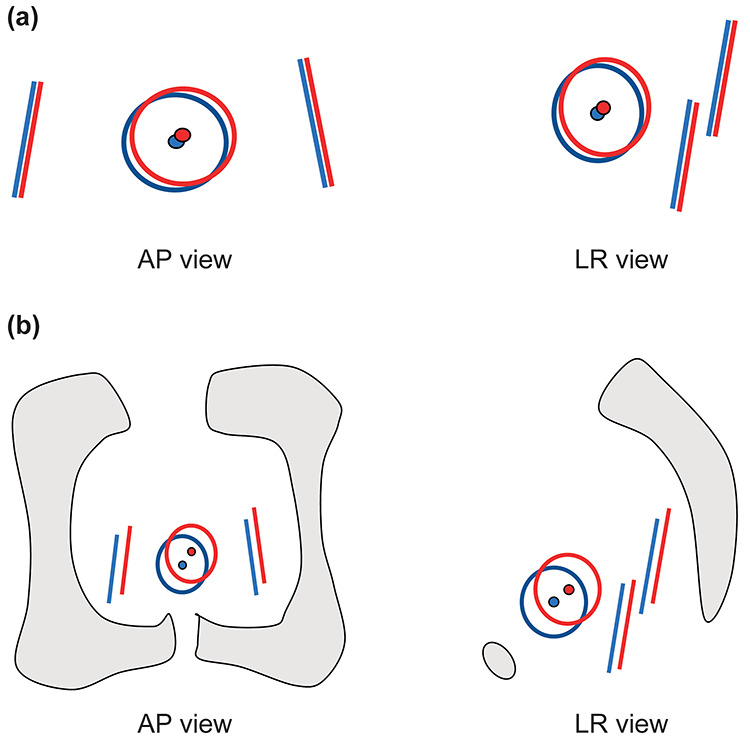
Schematic diagram of the marker/bone matching method. Large circle shows the prostate contouring, small circle shows the prostate centroid, and straight lines show two gold markers. Blue represents the reference CT scan, and red represents an additional weekly CT scan for analysis. Gray represents the pelvic bone. (**a**) Gold marker matching aligned the upper and lower points of each gold marker on the left and right between the reference CT scan and an additional weekly CT scan (AP/LR view). (**b**) Pelvic bone matching aligned the pelvic bony anatomy between the reference CT scan and an additional weekly CT scan (AP/LR view).

A computed tomography (CT) scan (SOMATOM Sensation Open; Siemens Medical Solutions, Erlangen, Germany) was taken 1 month after LDR brachytherapy and used as a reference image. Axial CT images of the pelvic area were taken at 3-mm thickness and 3-mm intervals. Additional CT scans were performed weekly for 4–5 weeks during the irradiation period. The immobilization device was not used for CT scans. These weekly images were taken immediately after irradiation, so they could be considered nearly the same condition (e.g. bladder filling) as the actual treatment. In this study, all imaging information was transferred to a treatment planning system (Pinnacle Imaging Systems, version 9.2, Belmont, CA, USA). The prostate was contoured based on each CT scan by a radiation oncologist, and the prostate centroid was the calculated 3D center of mass of each patient’s prostate. The relative position error of the prostate centroid was calculated and analyzed based on the reference CT scan. Gold marker matching aligned the upper and lower points of each gold marker on the left and right (Fig. 3a). Pelvic bone matching was conducted by aligning with the pelvic bony anatomy (i.e. obturator foramen and pubic symphysis) as shown in Fig. 3b. For a total of 175 CT images in 38 patients, the setup error was measured by the average values of the relative position displacement [ LR, AP, inferior–superior (IS) axis direction and 3D displacement] of the prostate centroid at the time of gold marker matching and pelvic bone matching. Then, the setup error between gold marker matching and pelvic bone matching was compared using the Wilcoxon signed-rank test. Rotational corrections were not included in the analyses. Analyses were carried out using SPSS version 22.0 (SPSS Inc., Chicago, IL, USA).

### Margin calculation

The setup margin was calculated from the relative position error of the prostate centroid at the time of marker and bone matching using Stroom’s (2Σ + 0.7σ) [21] and van Herk’s (2.5Σ + 0.7σ) [20] margin formulae, where Σ is the standard deviation (SD) of systematic error and σ is the SD of random error. Mean displacements and SD were calculated in the LR-, AP- and IS-axes and 3D displacement for each patient. Σ was calculated from the SD of all mean displacements and σ from the root mean square of all SD values [20, 21]. In this study, the setup margin in the interfractional error was calculated, but the intrafractional error was not included.

## RESULTS

### Displacement of prostate centroid

The clinical characteristics of the patients who participated in this study are shown in Table 1.

The shift in the prostate centroid due to marker matching was highest along the AP-axis (1.3 ± 2.6 mm) and lowest along the LR-axis (−0.024 ± 0.60 mm). The shift due to bone matching was highest along the AP-axis (1.7 ± 3.4 mm). When comparisons were made between marker and bone matching, the shift in the prostate centroid was lower in the LR- and AP-axes when marker matching was used. Overall, the 3D displacement was significantly lower for marker matching (2.7 ± 2.0 mm) than for bone matching (3.8 ± 2.6 mm, *P* < 0.01; Table 2).

**Table 2:** Shift in prostate centroid: marker matching compared with bone matching; values are given as the mean ± SD

	Marker matching (mm)	Bone matching (mm)	*P*
LR	−0.024 ± 0.60	0.34 ± 0.46	<0.01
AP	1.3 ± 2.6	1.7 ± 3.4	0.05
IS	−0.31 ± 1.7	0.16 ± 2.5	0.03
3D displacement	2.7 ± 2.0	3.8 ± 2.6	<0.01

### Margin calculation

Table 3 shows Σ and σ values in the LR-, AP- and IS-axes and 3D displacement with marker matching and bone matching. Using the margin formula 2Σ + 0.7σ for marker matching, the highest margin was 5.6 mm along the AP-axis and the 3D displacement was 4.3 mm. The corresponding values obtained using the margin formula 2.5Σ + 0.7σ were 6.8 mm along the AP-axis and 5.2 mm for the 3D displacement (Table 3).

**Table 3:** Setup margin with marker and bone matching; Σ is the SD of systematic error and σ is the SD of random error

Setup margin (mm)	LR	AP	IS	3D displacement
Marker matching				
Σ	0.54	2.4	1.6	1.8
σ	0.25	1.2	0.79	1.1
2Σ + 0.7σ	1.2	5.6	3.7	4.3
2.5Σ + 0.7σ	1.5	6.8	4.4	5.2
Bone matching				
Σ	0.36	3.2	2.2	2.1
σ	0.28	1.6	1.3	1.6
2Σ + 0.7σ	0.91	7.5	5.3	5.3
2.5Σ + 0.7σ	1.1	9.1	6.4	6.4

The highest margin was also observed along the AP-axis (7.5 mm) with bone matching when the margin formula 2Σ + 0.7σ was used. The 3D displacement was 5.3 mm using this formula. The corresponding values obtained using the margin formula 2.5Σ + 0.7σ were 9.1 mm along the AP-axis and 6.4 mm for the 3D displacement (Table 3).

## DISCUSSION

The use of image guidance is effective as a validation tool for position verification during EBRT. IGRT is increasingly used in clinical practice, although its use in EBRT following LDR brachytherapy has not been extensively explored in patients with prostate cancer. Furthermore, there is little understanding regarding the placement of gold markers in the surrounding tissue of the prostate as an imaging guide during EBRT.

The results outlined in this paper suggest that the implantation of gold markers for monitoring positioning errors in EBRT following LDR brachytherapy provides a better estimate than that attained using bone anatomy. The relative error of the prostate centroid was smaller in gold marker matching than in bone matching. The study also suggests that the optimal setup margin is lower with the implantation of gold markers than with bone matching. However, the setup error is relatively higher with the implantation of gold markers in the surrounding tissue of the prostate than with implantation of gold markers in the prostate compared with that reported in the literature [1, 2, 8, 9, 14–16, 22–28]. When placed in the surrounding tissue of the prostate, markers can move relative to the prostate in contrast to placement inside the prostate. Filling of the rectum, such as the buildup of flatulence, may also cause a shift of the prostate that does not always synchronize with marker movement. In these cases, position matching for the prostate may be a less effective method than placement of markers inside the prostate. However, the results obtained in this study show that gold marker matching is better than pelvic bone matching because gold markers were placed adjacent to the prostate.

Based on coverage probability matrices and dose–population histograms, which are used to describe the specific choice of a margin on a target group of patients, Stroom [21] proposed that a margin of 2Σ + 0.7σ ensures that, on average, 99% of the clinical target volume (CTV) receives ≥95% of the prescribed dose. Van Herk [20] proposed that a margin of 2.5Σ + 0.7σ ensures that 90% of the patients in the population receive a minimum cumulative CTV dose of at least 95% of the prescribed dose. In the present study, the setup margins of 3D displacement of the prostate centroid owing to marker matching were 4.3 mm (calculated by 2Σ + 0.7σ) and 5.2 mm (calculated by 2.5Σ + 0.7σ), lower than those obtained with bone matching; these margins are acceptable with the use of high-precision techniques. As the target volume is a direct function of the setup margin, the approach used in this study helped reduce the possibility of inadvertently irradiating the normal tissue surrounding the tumor, which may increase the risk of morbidity. By accurately irradiating the target, the dose to the target (e.g. prostate) is increased, and the dose to the surrounding organs at risk (e.g. rectum and bladder) is decreased.

In the present study, compared with pelvic bone matching, the accuracy of position matching was better for marker matching. In another study [6], IGRT was shown to be associated with an improvement in biochemical tumor control among high-risk patients as well as with a lower rate of late urinary toxicity compared with non-IGRT. These results led to an improvement in biochemical tumor control and a decrease in urinary adverse events. Kok *et al*. also demonstrated decreases in gastrointestinal and urinary adverse events [7]. They showed that despite dose escalation, the use of fiducial marker IGRT (FMIGRT) in radical radiotherapy for prostate cancer significantly reduced the incidence of gastrointestinal toxicity and the duration of late genitourinary toxicity compared with conventional non-FMIGRT techniques. Therefore, we can expect an improvement in the biochemical control rate in prostate cancer as well as a decrease in intestinal and urinary tract-related adverse events if setup errors were reduced.

In the present study, only two markers were used for the 3D positional correction. Matching was possible using the four craniocaudal points of each gold marker. Using three markers would have increased the invasiveness of the procedure and increased the cost. As it is a 4-point matching method, using two markers allows sufficient 3D positional correction [29]. There were minimal complications in this study, and there were no cases of infection as a result of the seed treatment. A small amount of bleeding was noticed in some cases, but it was not severe enough to require intervention.

This study had several limitations. Patients were evaluated using CT only once a week rather than after every irradiation session. In our hospital, daily IGRT is performed with gold markers in Linac MV imaging (Fig. 1). Since we cannot use kV cone-beam CT, the prostate is not identifiable. Therefore, to see the deviation of the prostate position, it is necessary to perform another diagnostic CT separately. Prostate contouring with CT was difficult, and there were uncertainties potentially caused by contouring errors on the CT scan. However, inter-observer variability in prostate contouring was possibly limited in our study because the prostate was contoured only by one expert radiation oncologist. Only the interfractional position error between irradiations was evaluated, whereas the intrafractional error during each irradiation was not evaluated. Some movement of the gold markers was observed between each irradiation. As the patient follow-up period was short, long-term outcomes, such as the biochemical control rates and intestinal and urinary tract adverse events, were unknown. A longer follow-up period for future studies is recommended.

Once the techniques described in this study are firmly established, we envisage delivery of precise and accurate radiotherapy for patients with prostate cancer, improvement in the biochemical control rate, and a reduction in adverse events experienced by patients. Future studies with greater statistical power should be undertaken to evaluate the margin errors described in this study. Furthermore, the results may be extended to other conditions, such as lung or liver cancer, in which markers are placed in the vicinity of tumors rather than in the tumor itself.

In conclusion, this study revealed reduced setup errors during EBRT following LDR brachytherapy by marker matching compared with pelvic bone matching. In addition, the setup margin and 3D displacement were lower with marker matching than with pelvic bone matching.
